# Expression of *TaWRKY44*, a wheat *WRKY* gene, in transgenic tobacco confers multiple abiotic stress tolerances

**DOI:** 10.3389/fpls.2015.00615

**Published:** 2015-08-11

**Authors:** Xiatian Wang, Jian Zeng, Ying Li, Xiaoli Rong, Jiutong Sun, Tao Sun, Miao Li, Lianzhe Wang, Ying Feng, Ruihong Chai, Mingjie Chen, Junli Chang, Kexiu Li, Guangxiao Yang, Guangyuan He

**Affiliations:** The Genetic Engineering International Cooperation Base of Chinese Ministry of Science and Technology, Key Laboratory of Molecular Biophysics of Chinese Ministry of Education, College of Life Science and Technology, Huazhong University of Science and TechnologyWuhan, China

**Keywords:** abiotic stress tolerance, antioxidant systems, ROS elimination, stress-associated gene, wheat, WRKY

## Abstract

The WRKY transcription factors have been reported to be involved in various plant physiological and biochemical processes. In this study, we successfully assembled 10 unigenes from expressed sequence tags (ESTs) of wheat and designated them as *TaWRKY44*–*TaWRKY53*, respectively. Among these genes, a subgroup I gene, *TaWRKY44*, was found to be upregulated by treatments with PEG6000, NaCl, 4°C, abscisic acid (ABA), H_2_O_2_ and gibberellin (GA). The TaWRKY44-GFP fusion protein was localized to the nucleus of onion epidermal cells, and TaWRKY44 was able to bind to the core DNA sequences of TTGACC and TTAACC in yeast. The N-terminal of TaWRKY44 showed transcriptional activation activity. Expression of *TaWRKY44* in tobacco plants conferred drought and salt tolerance and transgenic tobacco exhibited a higher survival rate, relative water content (RWC), soluble sugar, proline and superoxide dismutase (SOD) content, as well as higher activities of catalase (CAT) and peroxidase (POD), but less ion leakage (IL), lower contents of malondialdehyde (MDA), and H_2_O_2_. In addition, expression of *TaWRKY44* also increased the seed germination rate in the transgenic lines under osmotic stress conditions while exhibiting a lower H_2_O_2_ content and higher SOD, CAT, and POD activities. Expression of *TaWRKY44* upregulated the expression of some reactive oxygen species (ROS)-related genes and stress-responsive genes in tobacco under osmotic stresses. These data demonstrate that TaWRKY44 may act as a positive regulator in drought/salt/osmotic stress responses by either efficient ROS elimination through direct or indirect activation of the cellular antioxidant systems or activation of stress-associated gene expression.

## Introduction

As sessile organisms, plants are easily affected by various environmental stresses, including biotic and abiotic stresses. Abiotic stress conditions cause a devastating influence on plant yield under field conditions (Suzuki et al., 2014), with drought, salinity and low temperature as three of the most vital problems for agriculture because these abiotic stresses limit the plant from exhibiting its full genetic potential (Zhu, 2002). Plants have evolved complex mechanisms to increase their tolerance to these abiotic stresses through both physical adaptations and molecular and cellular changes in response to these stresses (Knight and Knight, 2001). To date, extensive research has focused on identifying key regulation components, including signal transduction receptors or effectors (Shen et al., 2006; Park et al., 2009), transcription factors (Dubos et al., 2010; Lata and Prasad, 2011; Chen et al., 2012) and various functional proteins related to physiological and metabolic responses under drought or salt stresses (Yonamine et al., 2004; Wang et al., 2011). Through binding to the *cis*-acting elements in the promoters of stress-related genes, transcription factors play vital roles in stress responses (Nakashima et al., 2009). Among them, plant WRKY transcription factors, one of the largest families of transcriptional regulators in plants, have been proven to play crucial roles in response to various stresses. After the initial reports on WRKY transcription factors (Ishiguro and Nakamura, 1994), three members (WRKY1, WRKY2, and WRKY3) were identified from parsley (*Petroselinum crispum*) and the name WRKY (pronounced “worky”) was coined (Rushton et al., 1996).

WRKY family proteins are transcription factors that are characterized by a conserved DNA-binding WRKY domain. The WRKY domain consists of 60 amino acid residues at the N-terminal and an atypical zinc-finger structure at the C-terminal. It was reported that the conserved WRKY amino acid sequences have variants, including WRRY, WSKY, WKRY, WVKY, or WKKY, in some WRKY proteins (Xie et al., 2005). The zinc-finger structure is either Cx_4–5_Cx_22–23_HxH or Cx_7_Cx_23_HxC (Rushton et al., 2010). The WRKY transcription factors were further divided into three groups based on the number of WRKY domains (two domains in Group I proteins and one in the others) and the structure of their zinc fingers (C2H2 in Group II proteins and C2HC in Group III proteins) (Eulgem et al., 2000). The WRKY factors have high binding affinity to the TTGACC/T promoter element sequence called the W-box sequence, which is common to numerous defense-associated genes. The TGAC core sequence of the W-box elements is important for WRKY binding, as demonstrated by numerous binding experiments (Ulker and Somssich, 2004; Ciolkowski et al., 2008).

Major advances in WRKY transcription factor function research have occurred over the past 20 years (Rushton et al., 2010). A massive amount of evidence has demonstrated that WRKY transcription factors participate in numerous physiological processes, including pathogen defense (Dong et al., 2003), sugar signaling (Sun et al., 2003), senescence (Zhou et al., 2011), trichome development (Johnson et al., 2002), root growth (Ren et al., 2010), and hormone signaling (Mao et al., 2007; Jiang et al., 2014). In recent years, the physiological functions of WRKYs in abiotic stress have also been reported (Zhou et al., 2008; Luo et al., 2013). However, compared with the extensive progress on biotic stresses, the functional understanding of WRKY proteins in abiotic stress is limited (Niu et al., 2012). As a staple crop, wheat production is constrained by multi-environmental stresses, such as drought, salinity and extreme temperature (Hu et al., 2012). Therefore, to improve the stress tolerance of wheat through genetic engineering, the detailed mechanisms of abiotic stress responses in wheat must be clarified. As a hexaploid plant, some studies have indicated that there are at least 200 *WRKY* genes in wheat (Okay et al., 2014; Satapathy et al., 2014), but to date, less than one-third of *WRKY* genes have been cloned and only a few of them have been functionally analyzed. Therefore, identification and functional analysis of WRKYs in wheat remain a challenge. Overexpression of some *WRKY* genes conferring tolerance to abiotic stresses through activating the antioxidant system has been reported in other species, such as rice and *Arabidopsis*, but there have been limited studies in wheat due to its complex and large genome. In this work, we successfully assembled 10 new unigenes from the ESTs of wheat. Previous work has identified 43 genes named *TaWRKY1*-*TaWRKY43* in wheat (Niu et al., 2012), in order to build a systematic naming system of *WRKY* genes in wheat, we designated these 10 genes as *TaWRKY44*-*TaWRKY53*, respectively. Among these genes, the expression of *TaWRKY44* in transgenic tobacco plants was shown to confer drought/salt/osmotic tolerance through direct or indirect activation of cellular antioxidant systems or stress-associated genes to eliminate ROS accumulation.

## Materials and methods

### Plant materials and stress treatments

Wheat (*Triticum aestivum* L. cv. Chinese Spring) seeds were sterilized and then germinated in sterile water and cultured in growth chambers (16 h light/8 h dark cycle at 25°C) for 10 days. For stress and signaling molecule treatments, uniform and healthy 10-day-old seedlings were steeped in and sprayed with sterile water, a 100 mM NaCl solution, a 20% PEG6000 solution, 100 μM ABA, 10 mM H_2_O_2_ and 5 μM GA and incubated under light for 24 h. Leaves from sterile water treatment were taken as a control. For organ expression analysis, roots, stems and leaves were collected from sterile seedlings, while pistils and stamens were collected from wheat plants in the growth chamber. Leaves were independently harvested at 0, 1, 3, 6, 12, and 24 h; immediately frozen in liquid nitrogen; and stored at −80°C until RNA extraction.

### Cloning and bioinformatic analysis of *TaWRKYs*

Total RNA from wheat seedlings was extracted using a RNAprep pure Plant Kit (DP432, Tiangen), and after the removal of genomic DNA by DNase I (Code No. 2212, Takara), 1 μg of total RNA was used to synthesize first-strand cDNA using a RevertAid First Strand cDNA Synthesis Kit (# K1691, Fermentas). The wheat ESTs were obtained from the DFCI wheat gene index database and NCBI to assemble 10 unigenes that were then amplified from wheat cDNA by PCR using specific primer pairs (Supplementary Table 1). The PCR products were purified by a TIANgel Midi Purification Kit (DP209, Tiangen), ligated to the pMD-18T plasmid (TakaRa) and then sequenced. After confirmation of the full-length sequence of *TaWRKY44*, a homology search was done in NCBI using BLASTp.

### Subcellular localization of TaWRKY44

The complete coding sequence of *TaWRKY44* without the stop codon was ligated into the pBI121-GFP vector after it was amplified by PCR using primer P1 (Supplementary Table 2) with *Xba*I and *Bam*HI restriction sites to create a fusion construct (pBI121-TaWRKY44-GFP). After confirmation by sequencing, pBI121-TaWRKY44-GFP was introduced into onion (*Allium cepa* L.) epidermal cells by particle bombardment (PDS-1000, Bio-Rad). pBI121-GFP was used as a control. After incubation at 25°C for 24 h, the tissue was stained with DNA-specific nuclear stain 4′,6-diamidino-2-phenylindole (DAPI) for 10 min. Fluorescence microscopy images were observed using a fluorescence microscope (Olympus FV500, http://www.olympus-global.com/).

### Analysis of transcriptional activation in yeast cells

A transcription activation assay was performed in yeast strain AH109 according to the Yeast Protocols Handbook (Clontech). The full length coding region and truncated fragments of *TaWRKY44* were generated by PCR using primers P2-P7 (Supplementary Table 2). The PCR products were cloned into the pGBKT7 vector using *Eco*RI and *Pst*I sites and were named pBD–TaWRKY44-N, pBD–TaWRKY44-NW1, pBD–TaWRKY44-NW2, pBD–TaWRKY44, pBD–TaWRKY44-W2C, and pBD–TaWRKY44-C, respectively. Plasmid pGBKT7 (pBD) was used as a negative control. These constructs were transformed into yeast strain AH109 by the lithium acetate-method. After confirmation by screening on selective medium plates without tryptophan (SD/-Trp) and colony PCR, the positive colonies were transfered onto the SD/-His plates with or without X-α-D-Galactosidase (X-α-gal), and the growth status of the yeast cells were photographed after incubating the plates for 3 d to evaluate the transcription activation activities.

### W-box binding assay of TaWRKY44 in yeast

A yeast one-hybrid assay was used to investigate whether TaWRKY44 binds to the W-box element. The full length coding region of *TaWRKY44* was generated by PCR using primer P8 (Supplementary Table 2). The PCR products were cloned into the pGADT7 vector using *Eco*RI and *Bam*HI sites to obtain pGADT7-TaWRKY44. A 18-bp oligonucleotide sequence containing three tandem repeat copies of the W-box element (5′-TTGACC-3′, the core sequence is underlined) was cloned into the pHIS2.0 vector using *Eco*RI and *Sac*I sites to obtain the reporter vector pHIS2-W-box. Similarly, vectors pHIS2-mW-box1-5 (pHIS2-mW1-5) were obtained by mutating the core TGAC sequence of the W-box elements to TGAT, CGAC, TAAC, TGGC, or AAAA (the mutated sites are underlined). Oligonucleotides were obtained by direct annealing using primers P9-P14 (Supplementary Table 2). Using the lithium acetate-method, pGAD-TaWRKY44 and pHIS2-(m) W-box were co-transformed into yeast strain Y187, while pHIS2/pGAD-TaWRKY44, pHIS2/ pGADT7 and pHIS2-W-box/pGADT7 were also co-transformed as negative controls. The DNA-protein interaction was evaluated according to the growth status of yeast cells cultured on SD/-His/-Leu/-Trp plates with 0, 30, and 60 mM 3-amino-1,2,4-triazole (3-AT) for 3 d.

### RT-PCR

After stress and signaling molecule treatments, the transcription levels of these 10 *WRKY* genes were monitored for 24 h using semi-quantitative RT-PCR. The specificity of the primers (Supplementary Table 1) used in RT-PCR was confirmed by agarose gel electrophoresis and sequencing. The cDNA was obtained following the procedures mentioned above. All of the reactions were performed for 30 cycles using TaKaRa DNA polymerase; *TaActin* or *NtActin* were used as internal controls.

### Real-time quantitative PCR (qRT-PCR)

To investigate the expression levels of *TaWRKY44* in response to various treatments in different wheat tissues, qRT-PCR was applied. Three biological replicates of cDNA prepared as mentioned above were used as the template for amplification. The qRT-PCR was carried out following the SuperReal PreMix Plus (SYBR Green, FP205, Tiangen) on a CFX Connect™ Optics Module (Bio-Rad) Real-Time PCR System. The PCR conditions were 95°C for 15 min followed by 40 cycles at 95°C for 10 s and 60°C for 30 s and 72°C for 32 s. The primers (Supplementary Table 1) used in qRT-PCR were designed based on sequence characterization to avoid the highly conserved WRKY domains, and the efficiency and specificity of the primers were first confirmed by agarose gel electrophoresis, validated by melting curve analysis using CFX Manager Software and sequencing of the amplified PCR products. The expression of the *TaActin* gene was used as an internal control. The 2^−ΔΔCt^ method was used to calculate the relative gene expression (Livak and Schmittgen, 2001).

### Plant transformation and generation of transgenic plants

To generate transgenic tobacco plants expressing TaWRKY44-GFP fusion protein, plasmids pBI121-TaWRKY44-GFP, under control of the *Cauliflower mosaic virus* 35S (*CaMV* 35S) promoter, and pBI121-GFP (VC) were transferred into *Agrobacterium tumefaciens* strain LBA4404, respectively. Transgenic tobacco plants were generated using the leaf disc transformation method according to Horsch et al. (1985). The seeds from T_0_ transgenic plants were harvested and sown on MS medium containing kanamycin (100 mg L^−1^), and the kanamycin-resistant T_1_ seedlings were confirmed by amplification of the *TaWRKY44* and *GFP* genes using primers P15-P16 (Supplementary Table 2). Three independent transgenic T_2_ line seedlings (OE-1, OE-7, and OE-35), almost all survived on MS medium containing 100 mg L^−1^ of kanamycin. The pBI121-GFP vector control line was used in the following experiments. Expression of *TaWRKY44* in three of the selected putative transgenic plants was examined by semi-quantitative RT-PCR using primers P16. Similarly, semi-quantitative RT-PCR expression analysis of *NtActin* using primer P17 (Supplementary Table 2), was used as an internal control.

### Stress tolerance assays of the wild-type (WT), vector control (VC), and *TaWRKY44* transgenic plants

Seed of WT, VC and three transgenic lines (OE-1, OE-7, OE-35) were surface sterilized with 75% (v/v) ethanol for 10 s and 10% (v/v) H_2_O_2_ for 10 min before they were sown on MS medium under a 16 h light/8 h dark cycle at 25°C for 2 weeks and then were transplanted into containers filled with a mixture of soil and sand (3:1) for 3 weeks with regular watering. Five-week-old plants with similar growth state were used in the following experiments. For drought/salt stress tolerance assays, 30 seedlings from each line were withheld from watering for 3 weeks before re-watering for 1 week or were irrigated with 400 mM NaCl for 3 weeks. After 3 weeks of drought/salt stresses, the survival rates were calculated and the leaves were collected to measure the RWC; IL; MDA, proline and soluble sugar contents; H_2_O_2_ accumulation; and antioxidant enzymes (SOD, POD, CAT) activities. For the osmotic stress tolerance assay, 2-week-old seedlings were transplanted to MS with mannitol (300 mM) and NaCl (200 mM) for 1 week, as described above. Leaves were used to measure ROS accumulation and CAT, SOD and POD activities. Fifty sterilized seeds from each line were sown on MS with mannitol (0, 150, or 300 mM) and NaCl (0, 100 or 200 mM) for 2 week, and the germination rates were scored daily.

### Analysis of proline, RWC, IL, MDA, soluble sugar, H_2_O_2_ accumulation and antioxidant enzyme activities

The proline content was measured by the ninhydrin reaction method. Proline was extracted from approximately 0.5 g of fresh leaves homogenized in 5 mL of 3% suphosalicylic acid and heated at 100°C for 10 min, and then, 2 mL of the extracted solution was added into 2 mL of acetic acid and 2 mL of 2.5% acid ninhydrin reagent and heated at 100°C for 30 min; the color of the solution turned to red. After cooling to room temperature, 4 mL of methylbenzene was added to the solution and incubated for 10 min after 30 s of shaking. The methylbenzene solution was used as a control to determine the optical density of the supernatant organic phase at 520 nm. The RWC and IL were measured according to previous reports (Deng et al., 2013; Hu et al., 2013), and the MDA content was determined according to the thiobarbituric acid (TBA)-based colorimetric method as described by Draper et al. (1993), with slight modifications. The soluble sugar content was examined by the phenol reaction method according to a previous study (Kong et al., 2011), with little modification. Approximately 0.2 g of fresh leaves were boiled in 5 mL of distilled water for 30 min for extraction and then diluted with distilled water to 10 mL. Two milliliters of the diluted solution was mixed with 1 mL of 9% phenol and 5 mL of concentrated sulfuric acid. After standing for 30 min, distilled water was used as a control to determine the optical density of the aqueous extract at 485 nm. A standard curve was drawn to calculate the soluble sugar content. The activity of three antioxidant enzymes, CAT, POD, and SOD, and the content of H_2_O_2_ were spectrophotometrically measured using four detection kits (A001, A007, A084, and A064, Jiancheng Bioengineering Institute) following the manufacturer's instructions.

### Analysis of the downstream genes regulated by TaWRKY44

The control lines (WT and VC) and the transgenic lines cultured on MS medium were transplanted to MS medium with mannitol (300 mM) and NaCl (200 mM) for 1 week. The total RNA of the leaves was extracted to synthesize cDNA. The expressions of the 14 selected stress-related genes were detected using qRT-PCR. The *NtActin* gene (P17) was used as the internal control. The sequences of the qPCR primers are listed in Supplementary Table 2 (P18–P31).

### Sequence and statistical analysis

Amino acid sequences were aligned by DNAMAN 8, and a phylogenetic tree was constructed using Mega 5.0. The data were analyzed in Excel, and the mean values ±SD were calculated from three independent experiments. Student's *t*-test was applied for the significant difference statistical analysis.

## Results

### Identification of *WRKY* genes in wheat

In this work, ten new *WRKY* genes were identified from wheat and designated as *TaWRKY44*-*TaWRKY53*, respectively according to previous studies, and the characteristics and GenBank accession numbers of the *WRKY*s are provided in Supplementary Tables 3, 6. Multiple alignments of the deduced amino acid sequences of *TaWRKY44*-*TaWRKY53* clearly showed that these proteins contained the conserved WRKY domain (Supplementary Figure 1). Phylogenetic analysis of these 10 *TaWRKY* genes compared to other *WRKY*s (The GenBank accession numbers showed in Supplementary Table 5) from various plants demonstrated that these 10 *WRKY* genes could be divided into three subgroups (Supplementary Figure 2). The expression patterns of the 10 *WRKY*s under abiotic stresses and plant hormone treatments were analyzed by RT-PCR (Supplementary Table 4). The results showed that *TaWRKY44* was upregulated by multiple stress treatments, and *TaWRKY44* was thus chosen for the further analysis of its role in abiotic stress responses. *TaWRKY44* cDNA is 1212 bp, with an 1113-bp open reading frame (ORF), and the deduced TaWRKY44 protein contains 370 amino acid residues with a predicted relative molecular mass of 40.39 kDa and isoelectric point of 8.43. Evolutionary relationship analysis showed that the TaWRKY44 protein contains two conserved DNA-binding domains (WRKY domain) and a zinc finger region, indicating that it belongs to Group I. BLASTp analysis revealed that the amino acid sequence of *TaWRKY44* had an 90% sequence identity with the putative WRKY transcription factor 4 (EMS63397.1) from *Triticum urartu* and an 87% sequence identity with putative WRKY transcription factor 4 (EMT16145.1) from *Aegilops tauschii*. These results indicated that TaWRKY44 is a member of the WRKY family from wheat.

### Expression pattern of *TaWRKY44* under various stress conditions

**Figure 1:**
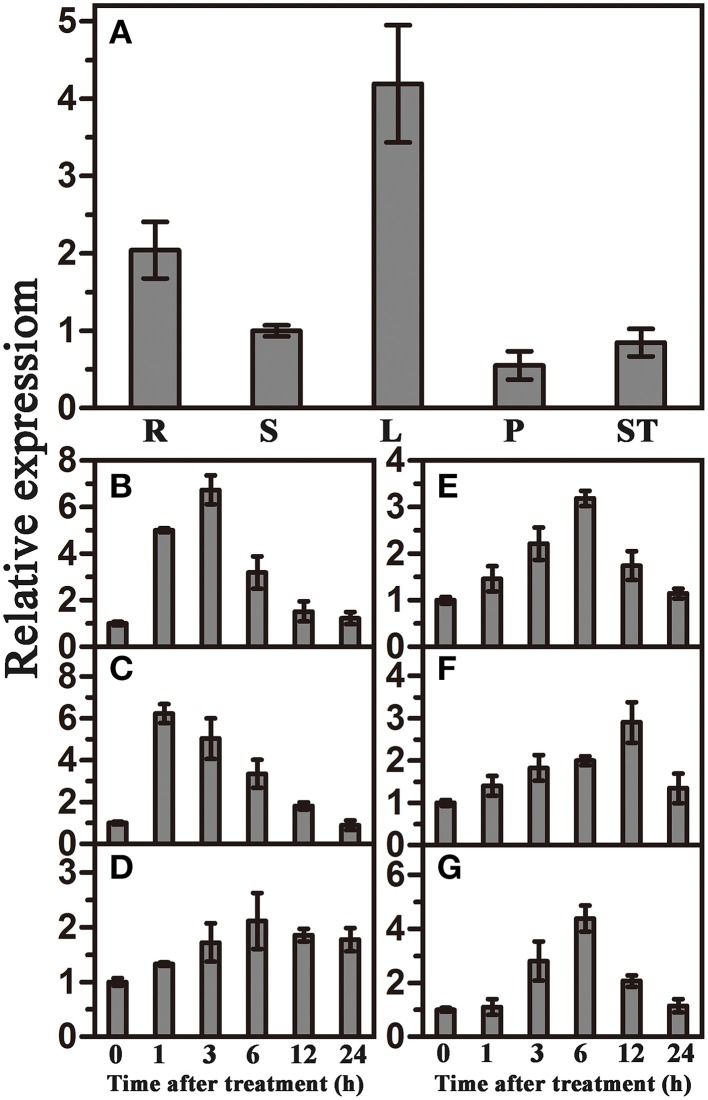
**Organ expression assay of ***TaWRKY44*** and expression profiles of ***TaWRKY44*** under treatments with PEG, NaCl, 4°C, ABA, GA, and H_2_O_2_ in wheat leaves. (A)** Organ expression assay of *TaWRKY44* in wheat. The organs (root, stem, leaf, pistil, and stamen) are represented by R, S, L, P, and ST, respectively. Expression analysis of *TaWRKY44* in 10-day-old wheat seedling leaves under different treatments by qRT-PCR, **(B)** 20% PEG6000; **(C)** 200 mM NaCl; **(D)** 4°C; **(E)** 100 μM ABA; **(F)** 10 mM H_2_O_2_; **(G)** 5 μM GA. For each assay, the expression level at time point 0 (for the stress assay) and stem (for the organ expression assay) was defined as 1.0, and the expression level at other time points and in other tissues was normalized accordingly. Error bars show the standard deviations for three independent replicates.

qRT-PCR was used to investigate the expression patterns of *TaWRKY44* in different tissues and under various abiotic stresses and signaling molecule treatments. The results showed that *TaWRKY44* was expressed in all tissues, with higher expression levels in leaves and roots and lower expression levels in stems, pistils and stamens (Figure 1A). *TaWRKY44* was obviously up-regulated after treatments with PEG and NaCl. During ABA and H_2_O_2_ treatments, the expression of *TaWRKY44* was gradually increased by 3.2-fold at 6 h and 2.9-fold at 12 h, respectively. Low temperature treatment led to a slight up-regulation, and GA treatment distinctly increased the expression of *TaWRKY44* (Figures 1B–G). On the other hand, with no treatment, the expression level of *TaWRKY44* had no obvious change (data not shown). These results demonstrated that the expression of *TaWRKY44* was up-regulated by PEG, NaCl, ABA H_2_O_2_, and GA treatments.

### Transcription activation activity of *TaWRKY44*

**Figure 2:**
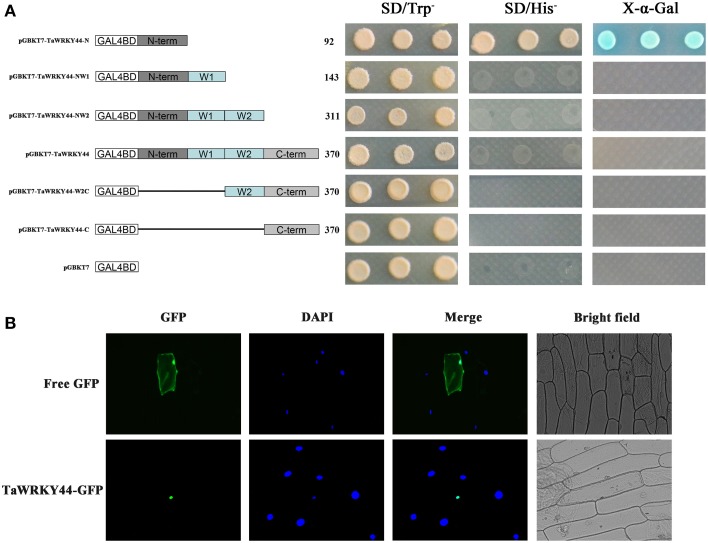
**Analysis of the transactivation activity and subcellular localization of TaWRKY44. (A)** Transactivation activity of the TaWRKY44 protein in yeast. Schematic diagrams of fused vectors illustrating the different portions of *TaWRKY44* that were fused to the yeast vector pGBKT7. Yeast strain AH109 was used in the transactivation activity analysis of *TaWRKY44*. The transformants were incubated on the SD/-Trp or SD/-His medium and subjected to X-α-gal assay. Three biological experiments produced similar results. **(B)** Subcellular localization of the TaWRKY44 protein in onion epidermal cells. The fusion protein TaWRKY44-GFP (pBI121- TaWRKY44-GFP) and GFP (pBI121-GFP) were transiently expressed in the onion epidermis using the bombardment method. Pictures were taken in bright and fluorescence fields after DAPI staining with fluorescence microscopy 24 h after bombardment. Three biological experiments were carried out, which produced similar results.

The yeast expression system was used to investigate whether TaWRKY44 possesses transcription activation activity. Yeast strain AH109 was transformed with fusion plasmids pGBKT7-TaWRKY44-N, pGBKT7-TaWRKY44-NW1, pGBKT7-TaWRKY44-NW2, pGBKT7-TaWRKY44, pGBKT7-TaWRKY44-W2C and pGBKT7-TaWRKY44-C, and pGBKT7 as a control. As shown in Figure 2A, the yeast cells transformed with pGBKT7- TaWRKY44- N grew well in His^−^ medium. Meanwhile, yeast cells transformed with other plasmids could only survive on SD/-Trp medium. The result of *LacZ* staining showed that the yeast cells transformed with pGBKT7-TaWRKY44-N turned blue in the presence of X-α-gal. These results indicated that the N-terminal region of TaWRKY44 has transcription activation activity, whereas the full-length TaWRKY44 appears to lack this activity.

### The TaWRKY44-GFP fusion protein is localized to the nucleus

The 35S-TaWRKY44-GFP plasmid was generated using the pBI121-GFP vector to investigate its subcellular distribution. Fluorescence imaging showed that the TaWRKY44-GFP fusion protein was localized exclusively to the nuclei of onion epidermal cells in a transient expression assay (Figure 2B). The control GFP was distributed through the cell. DAPI staining was used as a nuclear marker. The nuclear localization of TaWRKY44-GFP is consistent with its predicted function as a transcription factor.

### TaWRKY44 binds to the TGAC and TAAC core sequence

**Figure 3:**
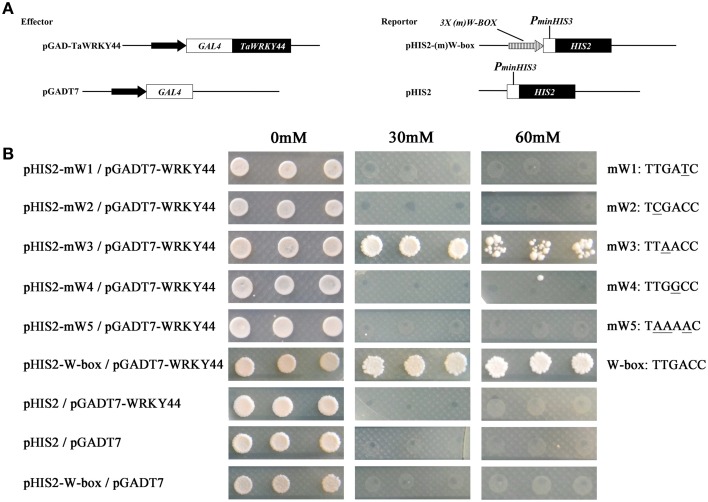
**Analysis of the W-box binding activity of TaWRKY44 using a yeast one-hybrid system**. **(A)** Schematic diagrams of the effector plasmids (pGAD-TaWRKY44 and pGADT7) and reporter plasmids (pHIS2-(m) W-box and pHIS2) used for the yeast one-hybrid assay. **(B)** Schematic diagrams of vector transformation combinations and the growth of yeast cells on SD/-His/-Leu/-Trp supplemented with (30 and 60 mM) or without 3-AT. The mW1-5 and W-box sequence indicated the core sequence of each vector. Three biological experiments produced similar results.

Plant WRKY proteins have high binding affinity to various W-box elements with the TGAC core sequence in the promoters of numerous defense-associated genes (Yu et al., 2001). A yeast one-hybrid system was used to evaluate the binding specificity between TaWRKY44 and the W-box (TTGACC/T) element. The full-length ORF of *TaWRKY44* was fused to the GAL4 activation domain of vector pGADT7, and the fused construct was co-transformed with pHIS2-W-box or the pHIS2-mW-box1-5 construct containing triple tandem repeats of the W-box and mutated W-box into yeast strain Y187 (Figure 3A). As shown in Figure 3B, all of the yeast cells transformed with the different combination of constructs described above could grow on SD/-Leu/-Trp/-His medium without 3-AT. However in the presence of 60 mM 3-AT, only the cells co-transformed with pGADT7-TaWRKY44 and pHIS2-W-box grew well, and the cells co-transformed with pGADT7-TaWRKY44 and pHIS2-mW-box3 (TTAACC) grew poorly, while others were completely inhibited. These results suggest that TaWRKY44 could strongly bind with the W-box core sequence TTGACC, even when the core sequence TTGACC was mutated to TTAACC, it could still weakly bind with it and activate the reporter gene in yeast.

### Generation of transgenic tobacco plants expressing *TaWRKY44*

**Figure 4:**
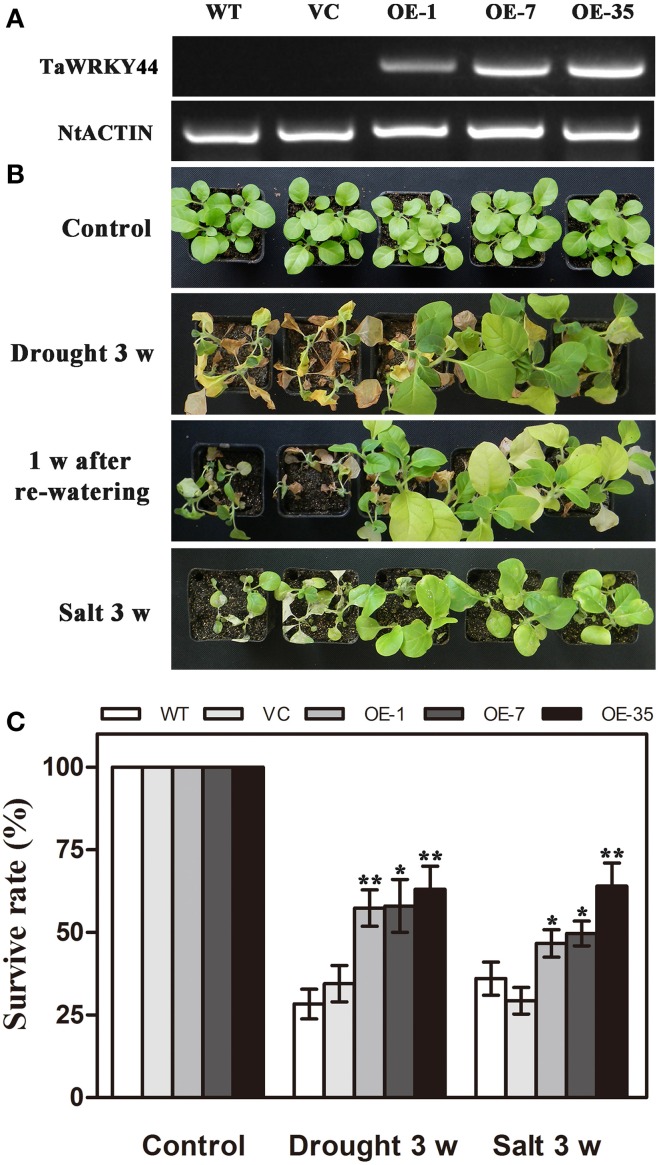
**Phenotype and survival rate of transgenic tobacco under drought and salt stress. (A)** Expression of *TaWRKY44* in the transgenic lines. **(B)** The photographs of WT, VC and transgenic lines after water withholding for 3 weeks, re-watering for 1 week and exposure to salt treatment (400 mM NaCl) for 3 weeks. **(C)** The survival rate of WT, VC and transgenic lines after drought and salt stress treatments. Data are the means ±SD calculated from three replicates. ^*^(*P* < 0.05) and ^**^(*P* < 0.01) indicate that the value in the transgenic lines is significantly different from that of the WT. Three biological experiments produced similar results.

As *TaWRKY44* was up-regulated by NaCl and PEG treatment, transgenic tobacco plants expressing TaWRKY44-GFP fusion protein were generated to examine the role of TaWRKY44 in salt and drought stress response, while the empty vector transformed into tobacco was served as a negative control. The ORF of *TaWRKY44* was ligated into the modified pBI121-GFP expression vector under the control of the *CaMV* 35S promoter. In total, 39 putative transgenic lines were confirmed by PCR. RT-PCR analysis showed that *TaWRKY44* mRNA was detected in transgenic plants, but not in the WT and VC, and *NtActin* was used as an internal control (Figure 4A). Three transgenic T_2_ lines (OE-1, OE-7, and OE-35) had a nearly 100% germination rate on MS medium containing 100 mg L^−1^ of kanamycin, which were thought to be homozygous transgenic lines, were used for further stress tolerance test.

### Expression of *TaWRKY44* enhances drought/salt tolerance in transgenic tobacco plants

To investigate whether transgenic expression of *TaWRKY44* was correlated with stress tolerance, the 5-week-old control lines (WT and VC) and transgenic line plants were subjected to drought/salt stress assays. For drought tolerance analysis, the 5-week-old plants were deprived of water for 3 weeks, followed by re-watering for 1 week. Leaf wilting was more evident in the WT and VC plants relative to the three transgenic lines after 3 weeks without watering (Figure 4B). For salt tolerance analysis, the 5-week-old plants were exposed to salt treatment (400 mM NaCl) for 3 weeks (Figure 4B). The survival rates of the transgenic lines were significantly higher than those of the control lines after drought/salt stresses (Figure 4C). These results indicated that the expression of *TaWRKY44* could enhance drought/ salt tolerance in transgenic tobacco.

### Expression of *TaWRKY44* increases RWC, proline and soluble sugar accumulation and decreases MDA and IL under drought/salt stresses

**Figure 5:**
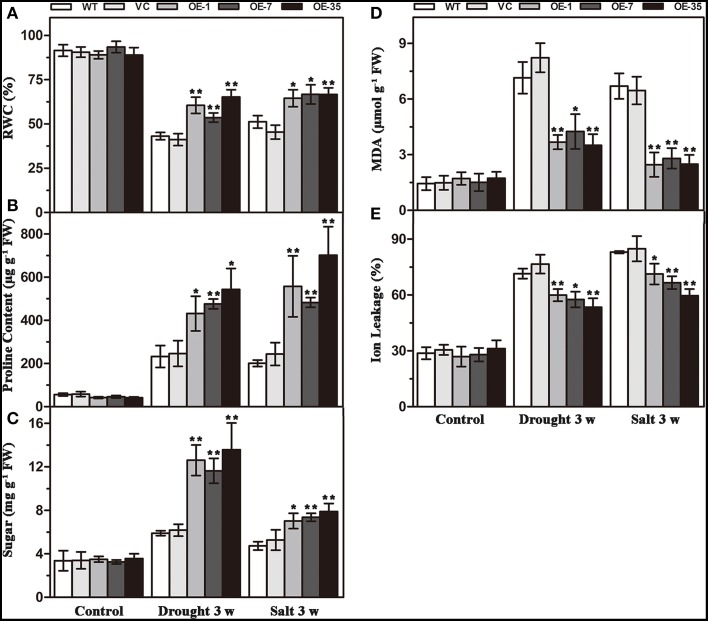
**Analysis of the physiological indices in the control (WT and VC) and transgenic lines (OE-1, OE-7 and OE-35) under normal and drought/salt conditions**. Five-week-old plants were subjected to drought and salt treatments for 3 weeks, then the leaves were sampled to assess the value of RWC **(A)**, proline content **(B)**, soluble sugar content **(C)**, MDA content **(D)**, and IL **(E)**. Data are the means ±SD calculated from three replicates. ^*^(*P* < 0.05) and ^**^(*P* < 0.01) indicate that the value in the transgenic lines is significantly different from that of the WT. Three biological experiments produced similar results.

That expression of *TaWRKY44* enhanced drought and salt tolerance led us to determine the effects of the physiological status caused by *TaWRKY44* expression. Result of RWC analysis, a credible evaluation of the plant water status, indicated that the activity of the plants under various environmental conditions was less reduced in transgenic lines after 3 weeks of drought/salt stress (Figure 5A). Proline is thought to play an important role as an osmotic-regulatory solute in plants subjected to hyperosmotic stresses, primarily through drought and soil salinity (Delauney and Verma, 1993). The proline content was higher in the transgenic lines after 3 weeks of drought/salt stress (Figure 5B). In addition, the soluble sugar levels exhibited a profile similar to that of proline (Figure 5C). These results showed that the transgenic lines possess more powerful resistance to hyperosmotic stresses compared to control lines. The MDA and IL levels, important indicators of membrane injury, were significantly lower in the transgenic lines relative to the control lines (Figures 5D,E), indicating that the WT and VC lines suffered from more severe membrane damage after drought/salt stress. Moreover, the transgenic lines showed no obvious difference with the control lines in these physiological indicators without stress treatment. These results demonstrated that the transgenic lines possess more powerful resistance to drought and salt stress.

### Expression of *TaWRKY44* increases antioxidant enzyme activity and decreases the H_2_O_2_ content under drought/salt stresses

Results of the MDA and IL levels indicated that the WT and VC lines suffered from more severe oxidative membrane damage after drought/salt stresses. Because enzymatic antioxidants could affect cellular ROS levels, we detected the levels of ROS and the activity of three significant antioxidant enzymes activities (SOD, POD, and CAT) in the leaves from the plants described above to further understand the relationships between enzymatic antioxidants and the influence of *TaWRKY44* expression on drought and salt stress tolerance. The results showed that after drought/salt treatments, the SOD, POD, and CAT activities in transgenic plants were significantly higher than those in the control plants; meanwhile, the H_2_O_2_ levels were lower in the transgenic plants (**Figure 7**). In addition, under normal growth condition, the SOD, POD, and CAT activities in transgenic plants were slightly higher than in the control plants, but there is no obvious difference in the H_2_O_2_ levels between the transgenic plants and the control plants. These results indicated that expression of *TaWRKY44* could influence the ROS levels by enhancing three significant antioxidant enzyme activities in the antioxidant system under drought/salt stress.

### Expression of *TaWRKY44* enhances osmotic tolerance in transgenic tobacco plants

**Figure 6:**
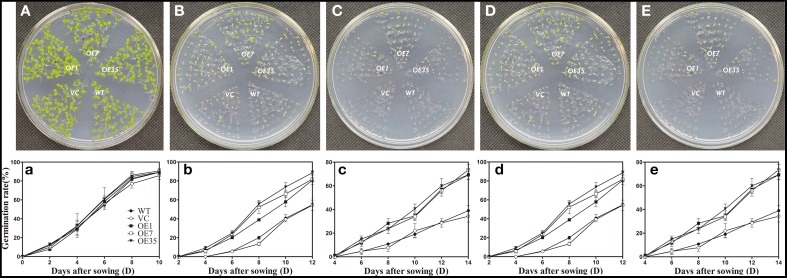
**Expression of ***TaWRKY44*** in tobacco increases the germination rate under osmotic stress on MS medium containing mannitol and NaCl**. The control (WT and VC) and transgenic lines (OE-1, OE-7 and OE-35) were sown on MS medium containing no mannitol and NaCl **(A,a)**, 150 mM **(B,b)**, 300 mM **(C,c)** mannitol, and 100 mM **(D,d)**, 200 mM **(E,e)** NaCl, the germination rates were monitored for 2 weeks before taking photographs. Panels **(A–E)** are the photos of germination status on media after 2 weeks; **(a–e)** are the chart of germination rate calculated for 2 weeks. Data are the means ±SD calculated from three replicates. Three biological experiments produced similar results.

To examine the osmotic stress tolerance of the transgenic plants, the control and transgenic lines were sown on MS medium containing mannitol (0, 150, or 300 mM) and NaCl (0, 100, or 200 mM) and the germination rate was monitored for 2 weeks before taking photographs. The results showed that the germination rates of the transgenic plants were obviously higher than those of the control plants grown on MS medium containing mannitol (150 or 300 mM) and NaCl (100 or 200 mM), while the plants grown on the MS medium without mannitol and NaCl had no significant difference between the control lines and transgenic lines (Figure 6). These results indicated that expression of *TaWRKY44* enhanced osmotic tolerance during seed germination in transgenic tobacco plants.

### Expression of *TaWRKY44* decreases ROS accumulation and improves SOD and cat activities under osmotic stress

**Figure 7:**
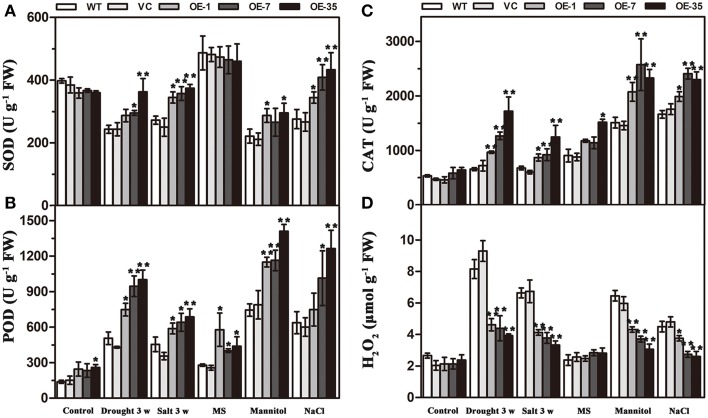
**Analysis of three antioxidant enzyme activities and H_2_O_2_ accumulation in the control (WT and VC) and transgenic lines (OE-1, OE-7 and OE-35) under normal and drought/salt/osmotic conditions**. Five-week-old plants were subjected to drought and salt treatments for 3 weeks. Two-week-old seedlings cultured on MS medium were transplanted to MS with mannitol (300 mM) and NaCl (200 mM) for 1 week, then the leaves were sampled to assess the SOD **(A)**, POD **(B)**, and CAT **(C)** activities and the H_2_O_2_ content **(D)**. Data are the means ±SD calculated from three replicates. ^*^(*P* < 0.05) and ^**^(*P* < 0.01) indicate that the value in the transgenic lines is significantly different from that of the WT. Three biological experiments produced similar results.

To further confirm the ability of transgenic tobacco plants to scavenge ROS, we detected the levels of ROS and three significant antioxidant activities in the leaves under osmotic stress. Two-week-old seedlings cultured on MS medium were transplanted to MS with mannitol (300 mM) and NaCl (200 mM) for 1 week. The results showed that without osmotic stress, there was no obvious difference in H_2_O_2_ accumulation and SOD, POD, and CAT activities between the control and transgenic plant seedlings; however, after mannitol and NaCl treatments, the SOD, POD, and CAT activities in the transgenic plants were significantly higher than in the control plants, but, the H_2_O_2_ levels were lower in the transgenic plants (Figure 7). These results indicated that the expression of *TaWRKY44* could influence the ROS levels by enhancing the activity of three significant antioxidant enzymes in the antioxidant system under osmotic stresses.

### TaWRKY44 regulates stress-responsive gene expressions under osmotic treatments

**Figure 8:**
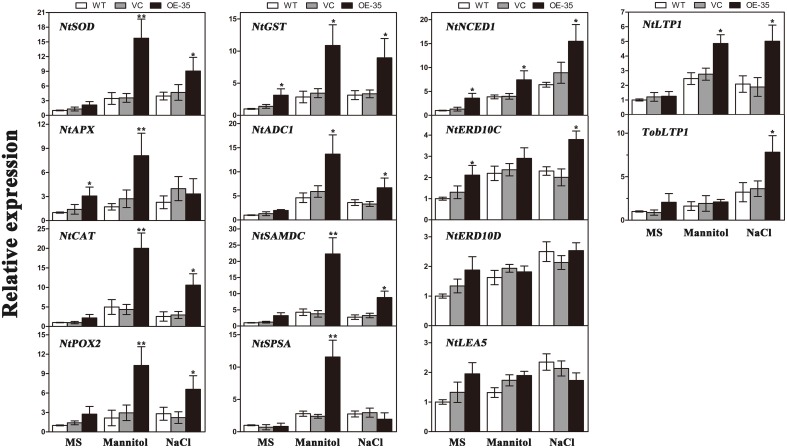
**Expression levels of ROS-related and stress-responsive genes in the WT, VC and the transgenic lines (OE-35) under normal and osmotic conditions analyzed qRT-PCR**. Two-week-old seedlings cultured on MS medium were transplanted to MS with mannitol (300 mM) and NaCl (200 mM) for 1 week. The tobacco leaves were then sampled to extract the total RNA to generate cDNA. Fourteen gene expression levels were detected; the *NtActin* gene was used as the internal control for normalization. Data are means ±SD calculated from three replicates. ^*^(*P* < 0.05) and ^**^(*P* < 0.01) indicate that the value in the transgenic line is significantly different from that of the WT. Three biological experiments produced similar results.

To gain further insights into the molecular mechanism underlying the enhanced drought/salt and osmotic resistance in transgenic tobacco plants, the expression levels of 14 ROS-related and stress-responsive genes were examined in the 2-week control and transgenic lines (OE-35) with or without 1 week of osmotic stress. We selected 14 genes listed below for this experiment: the genes encoding enzymes involved in ROS detoxification (*NtSOD, NtAPX, NtCAT, NtPOX*, and *NtGST*), enzyme genes for biosynthesis of polyamine (*NtADC1* and *NtSAMDC*), sucrose (*NtSPSA*), or abscisic acid (ABA; *NtNCED1*), stress response proteins (*NtERD10C, NtERD10D*, and *NtLEA5*) and lipid-transfer protein genes (*NtLTP1* and *TobLTP1*). Three-week-old seedlings, after 1 week of mannitol (300 mM) and NaCl (200 mM) treatment as described above, were used in this assay. Compared to the control plants, all of the stress-responsive genes analyzed were significantly upregulated in the transgenic line either when exposed to mannitol treatment, NaCl treatment or both, with the exception of *NtERD10D* and *NtLEA5* (Figure 8). These results demonstrated that the expression of *TaWRKY44* in tobacco enhances drought/salt/osmotic tolerance by inducing the expression of some ROS-related and stress-responsive genes.

## Discussion

Although it has been well documented that WRKY transcription factors are tightly connected with various plant defense mechanisms and respond to various adverse environmental conditions, functional studies have only been performed for a few WRKYs in wheat, and evidence for the relationships between WRKY proteins and abiotic stresses remains limited compared to biotic stresses. Meanwhile, the mechanisms of non-*WRKY* genes that enhance plant drought and salt stresses by either efficient ROS elimination through the activation of the cellular antioxidant systems or the activation of the stress-associated genes have been extensively reported in *Arabidopsis* (Moon et al., 2003; Luo et al., 2009), *Oryza sativa* (Ning et al., 2010; Kumar et al., 2012), *Poncirus trifoliate* (Huang et al., 2010, 2011), and wheat (Hu et al., 2012, 2013), but there is limited documentation of the function of WRKY proteins in wheat transcription processes.

In our present study, 10 new *TaWRKY* genes were identified from wheat. Phylogenetic analysis of these 10 *TaWRKY* genes compared to *WRKY*s from various plants demonstrated that these 10 *WRKY* genes could be divided into three subgroups (Supplementary Figure 2). Previous studies indicated that the Group I and group III *WRKY* genes were mainly involved in the abiotic stress response, while Group II *WRKY* genes were mainly involved in the response to biotic stresses, such as senescence, and a few studies have reported that an abiotic stress response, such as low Pi, was involved. BLASTp analysis revealed that *TaWRKY44* had 90% sequence identity with the putative WRKY transcription factor 4 (EMS63397.1) from *Triticum urartu* and 87% sequence identity with putative WRKY transcription factor 4 (EMT16145.1) from *Aegilops tauschii*. However, no functional analyses of these two genes have been published to date. *TaWRKY44* was upregulated by PEG, NaCl, cold (4°C), ABA, H_2_O_2_, and GA (Figure 1), which implied that *TaWRKY44* might play important roles in plant abiotic stress response. Transcriptional activation analysis demonstrated that only the N-terminal region of TaWRKY44 has transcriptional activation activity, whereas the full-length and C-terminal region of TaWRKY44 appears lacking this activity (Figure 2A). It is possible that TaWRKY44 needs additional posttranslational modifications to exhibit its full function. Additional modifications of transcription factors for stress tolerance have rarely been reported in crops (Tang et al., 2012). In a previous study, the soybean NAC transcription factor GmNAC20 appears to function as both a transcriptional repressor and transcriptional activator, and its activity as an activator or a repressor depends on a conformational change or its interactions with other regulatory proteins (Hao et al., 2011). The *Arabidopsis* transcription factor AREB1 activates expression of ABRE-dependent downstream genes through an ABA-induced modification of the AREB1 protein (Fujita et al., 2005), which is consistent with our results. Previous research indicated that phosphorylation is an important way to activate WRKY proteins (Ishihama et al., 2011), and potential phosphorylation sites of the TaWRKY44 protein were found using the NetPhos 2.0 Server (http://www.cbs.dtu.dk/services/NetPhos/, data not shown). This result implies a possible regulation of TaWRKY44 activity by phosphorylation *via* different protein kinases. Consistent with its putative role as a transcription factor, the TaWRKY44-GFP fusion protein was exclusively localized to the nuclei of onion epidermal cells in a transient expression assay similar to previous studies on other WRKY transcription factors (Figure 2B) (Lai et al., 2008). A yeast one-hybrid system was used to evaluate the binding specificity between TaWRKY44 and the W-box element (TTGACC/T), as in previous studies (Liu et al., 2013a; Zheng et al., 2013), and the results showed that TaWRKY44 had high affinity to the W-box core sequence TTGACC. Even when the core sequence was mutated to TTAACC, TaWRKY44 could still weakly bind to it and activate the reporter gene in yeast (Figure 3), suggesting that the G in TTGACC is not necessary for TaWRKY44 recognition. The induction of *TaWRKY44* expression under abiotic stresses promoted us to further clarify the function of TaWRKY44 in abiotic stress tolerance. Transgenic tobacco plants expressing TaWRKY44-GFP fusion protein were subjected to drought and salt stress treatments to examine the role of TaWRKY44 in salt and drought stress responses like some previous studies (Hu et al., 2013; Xu et al., 2014). The results indicated that the expression of *TaWRKY44* increased the survival rate under drought and salt stresses in transgenic tobacco (Figure 4). This result is consistent with previous work on other WRKYs. For instance, TaWRKY10 confers drought and salt tolerance in transgenic tobacco (Wang et al., 2013), expressing *TaWRKY2* confers salt and drought tolerance, and expression of *TaWRKY19* confers salt, drought and freezing tolerance in transgenic plants (Niu et al., 2012). The *abo3* mutant lost drought tolerance compared to the wild type and was hypersensitive to ABA in both seedling establishment and seedling growth in *Arabidopsis* (Ren et al., 2010). Constitutive expression of *BcWRKY46* in tobacco under the control of the *CaMV* 35S promoter reduced the susceptibility of transgenic tobacco to freezing, ABA, salt and dehydration stresses (Wang et al., 2012). On the basis of the phenotype analysis results, physiological and biochemical analysis was performed, and the results showed that the expression of *TaWRKY44* increased RWC, proline and soluble sugar accumulation and decreased MDA and IL under drought/salt stresses (Figures 5A–E). Because IL is an indicator of the severity of a membrane injury and MDA is a product of oxidative attack on membrane lipids (Moore and Roberts, 1998), we concluded that less oxidative damage occurred in transgenic plants under drought and salt stress. We detected the H_2_O_2_ levels in transgenic and control lines before and after drought and salt stress, and the results showed that the H_2_O_2_ levels in the transgenic lines were obviously lower than in the control lines after drought and salt stress (Figure 7D). These results indicated that the oxidative damage scavenging systems in transgenic plants might work more effectively compared with WT and VC. To detoxify stress-induced ROS, plants have evolved a complex antioxidant system (Miller et al., 2010). Plants possess very efficient enzymatic antioxidant defense systems to protect plant cells from oxidative damage by scavenging ROS (Gill and Tuteja, 2010). SOD provides the first line of defense against ROS by catalyzing the dismutation of O${}_{2}^{-}$ to oxygen and H_2_O_2_, which is then scavenged by the coordinated action of CAT and POD (Blokhina et al., 2003). The activities of three significant antioxidant enzymes (SOD, CAT, and POD) were assessed in transgenic lines and control lines before and after drought and salt stresses. The results indicated that the activities of three significant antioxidant enzymes (SOD, POD, and CAT) were higher than those in the control lines (Figures 6A–C). These results were similar to previous studies (Huang et al., 2010, 2011; Kong et al., 2011) and demonstrated that the antioxidant enzyme systems were activated to reduce the ROS levels in transgenic lines after drought and salt stresses. Moreover, germination assay was performed to examine the osmotic stress tolerance of the transgenic plants, and the germination rates of the transgenic plants were significantly higher than those of the control plants grown on MS medium containing 150/300 mM mannitol and 100/200 mM NaCl (Figure 6) and had lower H_2_O_2_ levels and improved antioxidant enzyme systems compared to controls under osmotic stress (Figure 7).

To gain further insights into the mechanisms of action of TaWRKY44 in drought/salt/osmotic stresses at the molecular level, the expression levels of 14 ROS-related and stress-responsive genes were tested under osmotic stress; these include genes encoding enzymes for ROS detoxification (*NtSOD, NtAPX, NtCAT, NtPOX*, and *NtGST*); enzymes involved in the biosynthesis of polyamine (*NtADC1* and *NtSAMDC*), sucrose (*NtSPSA*) or ABA (*NtNCED1*); stress-defensive proteins (*NtERD10C, NtERD10D*, and *NtLEA5*); and lipid-transfer protein genes (*NtLTP1* and *TobLTP1*). It was found that all of the stress-responsive genes analyzed were significantly upregulated in the transgenic lines either when exposed to mannitol (300 mM) or NaCl (200 mM) treatment or both, with the exception of *NtERD10D* and *NtLEA5*, compared to the control plants (Figure 8). The expression levels of the genes encoding three antioxidant enzymes were upregulated in the *TaWRKY44*-expressing lines with or without stress treatments (Figure 8, *NtSOD, NtCAT*, and *NtPOX2*), which is consistent with the results for the antioxidant enzyme activities described above. This could be an explanation for the antioxidant enzymes activities and the H_2_O_2_ contents in the transgenic lines and controls under drought/salt/osmotic stresses. Although the elaborate mechanism underlying the up-regulation of these antioxidant genes has not been clearly understood, previous studies have shown that the WRKY transcription factors could regulate the expressions of ROS-related genes in *Tamarix hispida* (Zheng et al., 2013), wheat (Niu et al., 2012; Wang et al., 2013), soybean (Luo et al., 2013), and cotton (Yan et al., 2014) under various stresses. On the other hand, two genes (*NtADC1* and *NtSAMDC*) related to the synthesis of polyamines, which are low-molecular-weight polycations that have been proven to be important stress molecules (Groppa and Benavides, 2008; Jang et al., 2009), were also induced in the transgenic lines relative to the control lines (Figure 8, *NtADC1* and *NtSAMDC*). This polyamine function (osmotic regulator or membrane stabilizer) in stress response could provide another explanation for the enhanced tolerance seen in the transgenic lines. Previous studies demonstrated that SPSA is critical in the synthesis of sucrose in plants and plays an essential role in plant osmotic pressure; NtNECD1 plays an essential role in ABA biosynthesis regulation (Huang et al., 2010); NtLEA5 and NtERD10 (C/D) belong to the LEA protein family that protects and stabilizes macromolecules and/or cellular structures during plant stress responses (Xiong and Zhu, 2002; Liu et al., 2013b); and NtLTP1 and TobLTP1 encode the lipid-transfer proteins, which are involved in plant response to ABA, cold, drought and salt stresses (Torres-Schumann et al., 1992; Hu et al., 2013). The up-regulation of these genes implies that these proteins may act as the intermediates between TaWRKY44 and the phenotype under drought/salt/osmotic stresses. These results demonstrated that the mechanism of enhanced drought/salt/osmotic tolerance in transgenic tobacco plants is the increase of the expression levels of some ROS-related and stress-responsive genes.

In conclusion, a wheat Group I *WRKY* gene, *TaWRKY44*, was upregulated by PEG, NaCl, ABA, and H_2_O_2_ treatments. Expression of *TaWRKY44* enhanced tolerance to drought, salt and osmotic in transgenic tobacco with increased RWC, proline and soluble sugar accumulation, decreased MDA and IL, improved antioxidant system and up-regulated transcription levels of ROS-related and stress responsive genes under various stresses. However, the elaborate mechanisms underlying these phenomena need to be clarified. Although whether these phenomena (compounds accumulation, changes in enzyme activity, genes expression etc.) were caused directly or indirectly by overexpressing *TaWRKY44* cannot be concluded in this study, but these changes were absolutely produced by overexpressing *TaWRKY44*. In our study, we found that full-length of TaWRKY44 has no transcriptional activation activity, it is suggested that TaWRKY44 needs additional posttranslational modifications or interactions with its cofactors to exhibit its full function. Therefore, it is possible that the genes encoding cofactors of TaWRKY44 were also induced by stress conditions. The induced expression of these genes, together with constitutive expression of *TaWRKY44*, resulted in up-regulation of these stress-related genes, and the increased tolerance of transgenic tobacco to drought/salt/osmotic stresses. In the future, it is necessary to identify if TaWRKY44 could function directly as transcription factor through binding to the upstream sequence of these genes, result of which will shed light on the mechanisms of TaWRKY44-mediated stress tolerance.

### Conflict of interest statement

The authors declare that the research was conducted in the absence of any commercial or financial relationships that could be construed as a potential conflict of interest.

## Supplementary material

Supplementary Table 1 **Primer sequences used for cloning wheat *WRKY* genes and expression analysis**.

Supplementary Table 2 **Primer sequences used for subcellular localization, vector construction, transgenic confirmation and expression analysis**.

Supplementary Table 3 **Characteristics of *TaWRKY44-TaWRKY53* in wheat (*T. aestivum* L. cv. Chinese Spring)**.

Supplementary Table 4 ***TaWRKY44-TaWRKY53* expression patterns under abiotic stresses and signaling molecule treatments in wheat (*T. aestivum* L. cv. Chinese Spring)**.

Supplementary Table 5 **The GenBank accession numbers of the WRKY proteins used for drawing phylogenetic tree**.

Supplementary Table 6 **The GenBank accession numbers of the *TaWRKY44-TaWRKY53***.

Supplementary Figure 1 **Multiple alignments of the deduced amino acid sequences of TaWRKY44-TaWRKY53**.

Supplementary Figure 2 **Phylogenetic analysis of TaWRKY domains from various plants**.

## Abbreviations

3-AT: 3-Amino-1, 2,4-triazole
ABA: Abscisic acid
*CaMV*: *Cauliflower mosaic virus*
CAT: Catalase
DAPI: 4'6-diamidino-2-phenylindole
GA: Gibberellin
GFP: Green fluorescent protein
IL: Ion leakage
LEA: Late embryogenesis abundant
MDA: Malonaldehyde
MS: Murashige and Skoog
OE: Overexpression
ORF: Open reading frame
POD: Peroxidase
QRT-PCR: Real-time quantitative PCR
ROS: Reactive oxygen species
RT-PCR: Reverse transcription–PCR
RWC: Relative water content
SOD: Superoxide dismutase
VC: Vector control
WT: Wild type.
