# Supplementary material for: Expression of TaWRKY44, a wheat WRKY gene, in transgenic tobacco confers multiple abiotic stress tolerances
Source: Front Plant Sci. 2015 Aug 11;6:615. doi: 10.3389/fpls.2015.00615 (PMC4531243; doi:10.3389/fpls.2015.00615)
Supplement: Supplementary Table 1 — Primer sequences used for cloning wheat WRKY genes and expression analysis. [file Table1.DOC]

**Supplementary Table 1. Primer sequences used for cloning wheat *WRKY* genes and expression analysis**

| **Gene Name** | **Primer Name** | **Sequences (5'- 3)**  **Forward Reverse** | |  |
| --- | --- | --- | --- | --- |
| *TaWRKY44* | *TaWRKY44* Full Length | gccccccttcgctcttctc | CAGCACACCAGAAATGGGCTAAT | |
| *TaWRKY45* | *TaWRKY45* Full Length | TTCCCTTCCCCTTTCGAG | CAGAAGCTAACCCAACAACAG | |
| *TaWRKY46* | *TaWRKY46* Full Length | TCCATGTCCGGATCAGCAGC | ATGCACATGCGCCCGTAGA | |
| *TaWRKY47* | *TaWRKY47* Full Length | TGTAGTTGCATGGCATCTTCGGG | TGGAACCACCGGAAAAAAAAGGA | |
| *TaWRKY48* | *TaWRKY48* Full Length | GGAAGACATGGACGAGCAGTGGA | GCTGGCAGATTTCCGGTCAAG | |
| *TaWRKY49* | *TaWRKY49* Full Length | CACGCCCTACTCTCTATGCCCTC | TGGCATCCAATACAAACATATTACCTTC | |
| *TaWRKY50* | *TaWRKY50* Full Length | GGGAGGAGATGGAGGAGAGGTGC | CCTGCGGAGACTTAGAGATAGCG | |
| *TaWRKY51* | *TaWRKY51* Full Length | CGCTCTCGCCGGTGTCAAACT | TGCTCCGTCGTTGGTGGCTAG | |
| *TaWRKY52* | *TaWRKY52* Full Length | TCGCAATGTCCTCCTACTCGTC | TCGCAATGTCCTCCTAC | |
| *TaWRKY53* | *TaWRKY53* Full Length | ACCAAGACAGCGAGCCAAGATC | CACCAAAAGGGGAAAGAAAGAAAA | |
| *TaWRKY44* | *TaWRKY44* RT-PCR and qRT-PCR | CCAACGGCGGTGATAACTACAT | GCTACTGGATGCTGCCTTCTG | |
| *TaWRKY45* | *TaWRKY45* RT-PCR | GGAGGCGCCGGCAGAGGATAACC | GGCCCGCAAACCCTGGAGGAG | |
| *TaWRKY46* | *TaWRKY46* RT-PCR | GCCGGAGGAGGGAGACGAGGA | ATGCACATGCGCCCGTAGACGT | |
| *TaWRKY47* | *TaWRKY47* RT-PCR | TGCCCCGACGTTTCCTCAGTTG | TGGAACCACCGGAAAAAAAAGGATT | |
| *TaWRKY48* | *TaWRKY48* RT-PCR | CGGCGAGTGGGCGAGGAGAACAG | GGGGCCAACGGTGACGCTGAC | |
| *TaWRKY49* | *TaWRKY49* RT-PCR | CGGCTGGCCACGGGAAGTTCA | TGCCAAATGACCCCCCTAACGATC | |
| *TaWRKY50* | *TaWRKY50* RT-PCR | GCGGCGCTGACAGAGGGGAGA | TTGGGTACTTGGCGCCGAGGA | |
| *TaWRKY51* | *TaWRKY51* RT-PCR | CGCTCTCGCCGGTGTCAAACTC | CCACTTGTAGCCGTCGTCGAGGA | |
| *TaWRKY52* | *TaWRKY52* RT-PCR | CGGCAGTGGCGCAAGGTGGT | GCCTCCTACCCCTTGCACGCACAG | |
| *TaWRKY53* | *TaWRKY53* RT-PCR | GCCGTCTCCAAGTTCCGCAAGGT | GCCCTTGGACACGCTGCCCTC | |
| *TaActin* | *Wheat Actin* | CTTGTATGCCAGCGGTCGAACA | CTCATAATCAAGGGCCACGTA | |
