# Supplementary material for: Expression of TaWRKY44, a wheat WRKY gene, in transgenic tobacco confers multiple abiotic stress tolerances
Source: Front Plant Sci. 2015 Aug 11;6:615. doi: 10.3389/fpls.2015.00615 (PMC4531243; doi:10.3389/fpls.2015.00615)
Supplement: Supplementary Table 2 — Primer sequences used for subcellular localization, vector construction, transgenic confirmation and expression analysis. [file Table2.DOC]

**Supplementary Table 2. Primer sequences used for subcellular localization, vector construction, transgenic confirmation** and expression analysis

| **Gene Name** | **Primer Name** | **Forward Reverse** | |  |
| --- | --- | --- | --- | --- |
| *TaWRKY44* | P1 | TCTAGAATGTCCCATCAGCAAGCGTT  (*Xba*I site is underlined) | ggATCCTTgTTATCTCATTTTCTTCTTTTA  (*Bam*HI site is underlined) | |
| *TaWRKY44* | P2 | gAATTCATgTCCCATCAgCAAgCgTT | CTgCAgATTgTACCCATCATCAgCAggT | |
| *TaWRKY44* | P3 | gAATTCATgTCCCATCAgCAAgCgTT | CTgCAggTggTTATgCTggCCTCTATA | |
| *TaWRKY44* | P4 | gAATTCATgTCCCATCAgCAAgCgTT | CTgCAgATggCTATgCTTgCCTTCAT | |
| *TaWRKY44* | P5 | gAATTCATgTCCCATCAgCAAgCgTT | CTgCAgTTATgTTATCTCATTTTCTTCT | |
| *TaWRKY44* | P6 | gAATTCTggCgCAAgTACgggC | CTgCAgTTATgTTATCTCATTTTCTTCT | |
| *TaWRKY44* | P7 | gAATTCgATgTgCCAgCAgCTAggAg  (*Eco*RI site is underlined from P2-P7) | CTgCAgTTATgTTATCTCATTTTCTTCT  (*Pst*I site is underlined from P2-P7) | |
| *TaWRKY44* | P8 | cggaattcATGTCCCATCAGCAAGCGTT  (*Eco*RI site is underlined) | cgggatcccTTATGTTATCTCATTTTCTT  (*Bam*HI site is underlined) | |
| *W-box* | P9 | aattcTTGACCTTGACCTTGACCgagct | cGGTCAAGGTCAAGGTCAAg | |
| *mW-box1* | P10 | aattcTTGAtCTTGAtCTTGAtCgagct | cGaTCAAGaTCAAGaTCAAg | |
| *mW-box2* | P11 | aattcTcGACCTcGACCTcGACCgagct | cGGTCgAGGTCgAGGTCgAg | |
| *mW-box3* | P12 | aattcTTaACCTTaACCTTaACCgagct | cGGTtAAGGTtAAGGTtAAg | |
| *mW-box4* | P13 | aattcTTGgCCTTGgCCTTGgCCgagct | cGGcCAAGGcCAAGGcCAAg | |
| *mW-box5* | P14 | aattcTaaaaCTaaaaCTaaaaCgagct  (*Eco*RI and *Sac*I sites are underlined  from P9-P14) | cGttttAGttttAGttttAg  (Mutated sites are lowercased writing from P9-P14) | |
| pBI121-TaWRKY44-GFP | P15 | AGGGCAGAAGGCAGCATCCAGTA | ATCCCTCAGGCATGGCGCTCTT | |
| pBI121-TaWRKY44-GFP | P16 | ATgAgTAAAggAgAAgAAC | TTATTTgTATAgTTCATCCA | |
| *NtActin* | P17 | CTATTCTCCGCTTTGGACTTGGCA | ACCTGCTGGAAGGTGCTGAGGGAA | |
| *NtERD10D* | P18 | GAGGACACGGCTGTACCAGT | GCGCCACTTCCTCTGTCTT | |
| *NtERD10C* | P19 | AACGTGGAGGCTACAGATCG | GTTCCTCTTGGGCATGAGTT | |
| *NtLEA5* | P20 | TTGAATCTGGGGTTTTGGTT | GGAAGCATTGACGAGCTAGG | |
| *NtNCED1* | P21 | AAGAATGGCTCCGCAAGTTA | GCCTAGCAATTCCAGAGTGG | |
| *NtLTP1* | P22 | GCAGAAGCCATAACCTGTGG | CAGTGGAAGGGCTGATCTTG | |
| *TobLTP1* | P23 | GGTTTTGTGCATGGTGGTGG | CTTAGAGCAGTCTGTGGAGG | |
| *NtPOX2* | P24 | CTTGGAACACGACGTTCCTT | TCGCTATCGCCATTCTTTCT | |
| *NtSOD* | P25 | CTCCTACCGTCGCCAAAT | GCCCAACCAAGAGAACCC | |
| *NtCAT* | P26 | AGGTACCGCTCATTCACACC | AAGCAAGCTTTTGACCCAGA | |
| *NtAPX* | P27 | CAAATGTAAGAGGAAACTCAGAGGA | CAGCCTTGAGCCTCATGGTACCG | |
| *NtGST* | P28 | CCCCTAGTTTGCTCCCTTCT | TTCTTAGCTGCCTCCTGCTC | |
| *NtSPSA* | P29 | GAATTCAGGCGCTTCGTTGTCA | ACCCCTAGTTTCTCCAGTGA | |
| *NtADC1* | P30 | CTTGCTGATTACCGCAATTTATC | TAGGATCAGCAGCCCCCATAGCC | |
| *NtSAMDC* | P31 | CATTCACATTACCCCGGAAG | AGCAACATCAGCATGCAAAG | |
