# Supplementary material for: Expression of TaWRKY44, a wheat WRKY gene, in transgenic tobacco confers multiple abiotic stress tolerances
Source: Front Plant Sci. 2015 Aug 11;6:615. doi: 10.3389/fpls.2015.00615 (PMC4531243; doi:10.3389/fpls.2015.00615)
Supplement: Supplementary Table 3 — Characteristics of TaWRKY44-TaWRKY53 in wheat (T. aestivum L. cv. Chinese Spring). [file Table3.DOC]

**Supplementary Table 3. Characteristics of *TaWRKY44*-*TaWRKY53* in wheat (*T. aestivum* cv Chinese Spring)**

| Gene Name | cDNA | | No. of Amino Acid Residues | N-Terminal | C-Terminal | WRKY Domain | Zinc Finger Motif | Sub Group |
| --- | --- | --- | --- | --- | --- | --- | --- | --- |
|  | Full length | Length (bp) |  |  |  |  |  |  |
| *TaWRKY44* | Y | 1212 | 370 | MSHQQAL | KEENEIT | WRKYGQK | C_2_H_2_ | Ⅰ |
| *TaWRKY45* | Y | 1682 | 522 | MADGEPE | AVPVQNN | WRKYGQK | C_2_H_2_ | Ⅰ |
| *TaWRKY46* | N | 794 | 263 | MSGSAAA | － | WRKYGQK | C_2_H_2_ | Ⅰor Ⅱ |
| *TaWRKY47* | Y | 944 | 299 | MASSGSA | DFFLFDP | WRKYGQK | C_2_HC | Ⅲ |
| *TaWRKY48* | Y | 1001 | 325 | MDEQWMI | SGRIPVA | WRKYGQK | C2H2 | Ⅱ |
| *TaWRKY49* | Y | 1263 | 380 | MASCGGA | GSSNIFL | WRKYGQK | C_2_HC | Ⅲ |
| *TaWRKY50* | Y | 895 | 291 | MEERCAL | LDNSRYL | WRKYGQK | C_2_HC | Ⅲ |
| *TaWRKY51* | Y | 724 | 223 | MAAVGAA | PPPGSLN | WRKYGKK | C2H2 | Ⅱ |
| *TaWRKY52* | Y | 618 | 199 | MSSYSSL | AQTRVDE | WRKYGKK | C2H2 | Ⅱ |
| *TaWRKY53* | Y | 1034 | 313 | MAVDLMG | SAGNGHV | WRKYGQK | C2H2 | Ⅱ |
