# Supplementary material for: Expression of TaWRKY44, a wheat WRKY gene, in transgenic tobacco confers multiple abiotic stress tolerances
Source: Front Plant Sci. 2015 Aug 11;6:615. doi: 10.3389/fpls.2015.00615 (PMC4531243; doi:10.3389/fpls.2015.00615)
Supplement: Supplementary Table 4 — TaWRKY44-TaWRKY53 expression patterns under abiotic stresses and signaling molecule treatments in wheat (T. aestivum L. cv. Chinese Spring). [file Table4.DOC]

**Supplementary Table 4. *TaWRKY44*-*TaWRKY53* expression patterns under abiotic stresses and signaling molecule treatments in wheat (*T aestivum* cv Chinese Spring)**

| Genes | PEG6000 | | 4℃ | | NaCl | | ABA | | GA | | H_2_O_2_ | | Light | |
| --- | --- | --- | --- | --- | --- | --- | --- | --- | --- | --- | --- | --- | --- | --- |
| *TaWRKY44* | | **↑** | | **↑** | | **↑** | | **↑** | | **↑** | | **↑** | | **－** |
| *TaWRKY45* | | **－** | | **－** | | **－** | | **↑** | | **↑** | | **－** | | **↑** |
| *TaWRKY46* | | **↑** | | **↑** | | **↑** | | **↑** | | **↑** | | **↑** | | **↑** |
| *TaWRKY47* | | **↑** | | **－** | | **↑** | | **－** | | **↑** | | **↑** | | **↗** |
| *TaWRKY48* | | **↙** | | **↗** | | **－** | | **－** | | **－** | | **－** | | **↑** |
| *TaWRKY49* | | **↑** | | **－** | | **↑** | | **↑** | | **↑** | | **↑** | | **－** |
| *TaWRKY50* | | **－** | | **↑** | | **↑** | | **↑** | | **↑** | | **↑** | | **↑** |
| *TaWRKY51* | | **↑** | | **↑** | | **↑** | | **↑** | | **↑** | | **↑** | | **↗** |
| *TaWRKY52* | | **↑** | | **↑** | | **↑** | | **↑** | | **↑** | | **↑** | | **－** |
| *TaWRKY53* | | **－** | | **↑** | | **↗** | | **↑** | | **↑** | | **↓** | | **－** |

Three biological experiments were performed by RT-PCR with independent RNA samples and produced similar results.

**↑** indicates up regulation in gene expression.

**↓** indicates down regulation in gene expression.

**↗**indicates slightly up regulation in gene expression.

**↘**indicates slightly down regulation in gene expression.

**－**indicates no significant changes in gene expression.
