# Supplementary material for: Expression of TaWRKY44, a wheat WRKY gene, in transgenic tobacco confers multiple abiotic stress tolerances
Source: Front Plant Sci. 2015 Aug 11;6:615. doi: 10.3389/fpls.2015.00615 (PMC4531243; doi:10.3389/fpls.2015.00615)
Supplement: Supplementary Table 5 — The GenBank accession numbers of the WRKY proteins used for drawing phylogenetic tree. [file Table5.DOC]

**Supplementary Table 5. The GenBank accession numbers of the WRKY proteins used to construct phylogenetic tree**

| Genes | GenBank accession No. | WRKY subgroups |
| --- | --- | --- |
| *AtWRKY20* | NM_118798.1 | Ⅰ |
| *OsWRKY30* | DQ298180.1 | Ⅰ |
| *AtWRKY33* | NM_129404.3 | Ⅰ |
| *BdWRKY4* | XM_010235097.1 | Ⅰ |
| *OsWRKY1* | AF193802.2 | Ⅰ |
| *AtWRKY25* | NM_128578,.3 | Ⅰ |
| *GmWRKY21* | DQ322691.1 | Ⅱ-c |
| *GmWRKY53* | DQ322693.1 | Ⅱ-c |
| *GmWRKY54* | DQ322698.1 | Ⅱ-c |
| *PtWRKY23* | EF051079.1 | Ⅱ-c |
| *AtWRKY18* | NM_119329.3 | Ⅱ-a |
| *AtWRKY60* | NM_128058.3 | Ⅱ-a |
| *AtWRKY40* | NM_106732.3 | Ⅱ-a |
| *AtWRKY61* | NM_101743.4 | Ⅱ-b |
| *AtWRKY9* | NM_105485.3 | Ⅱ-b |
| *AtWRKY47* | NM_116402.3 | Ⅱ-b |
| *AtWRKY6* | NM_104910.2 | Ⅱ-b |
| *AtWRKY31* | NM_118328.3 | Ⅱ-b |
| *AtWRKY22* | NM_116355.2 | Ⅱ-e |
| *AtWRKY29* | NM_118486.5 | Ⅱ-e |
| *AtWRKY14* | NM_102802.1 | Ⅱ-e |
| *AtWRKY35* | NM_129036.2 | Ⅱ-e |
| *AtWRKY39* | NM_111339.2 | Ⅱ-d |
| *AtWRKY7* | NM_118557.3 | Ⅱ-d |
| *GmWRKY13* | DQ322694.1 | Ⅱ-d |
| *AtWRKY17* | NM_128018.2 | Ⅱ-d |
| *AtWRKY46* | NM_130204.2 | Ⅲ |
| *AtWRKY54* | NM_129637.2 | Ⅲ |
| *AtWRKY70* | NM_115498.3 | Ⅲ |
| *AtWRKY41* | NM_117177.2 | Ⅲ |
| *TcWRKY53* | EF053036.1 | Ⅲ |
| *AtWRKY53* | NM_118512.3 | Ⅲ |
