# Supplementary material for: Expression of TaWRKY44, a wheat WRKY gene, in transgenic tobacco confers multiple abiotic stress tolerances
Source: Front Plant Sci. 2015 Aug 11;6:615. doi: 10.3389/fpls.2015.00615 (PMC4531243; doi:10.3389/fpls.2015.00615)
Supplement: Supplementary Table 6 — The GenBank accession numbers of the TaWRKY44-TaWRKY53. [file Table6.DOC]

**Supplementary Table 6. The GenBank accession numbers of the *TaWRKY44*-*TaWRKY53***

| Genes | GenBank accession No. |
| --- | --- |
| *TaWRKY44* | KR827395 |
| *TaWRKY45* | KR827396 |
| *TaWRKY46* | KR827397 |
| *TaWRKY47* | KR827398 |
| *TaWRKY48* | KR827399 |
| *TaWRKY49* | KR827400 |
| *TaWRKY50* | KR827401 |
| *TaWRKY51* | KR827402 |
| *TaWRKY52* | KR827403 |
| *TaWRKY53* | KR827404 |
