# Supplementary figures and images for: Expression of TaWRKY44, a wheat WRKY gene, in transgenic tobacco confers multiple abiotic stress tolerances
Source: Front Plant Sci. 2015 Aug 11;6:615. doi: 10.3389/fpls.2015.00615 (PMC4531243; doi:10.3389/fpls.2015.00615)

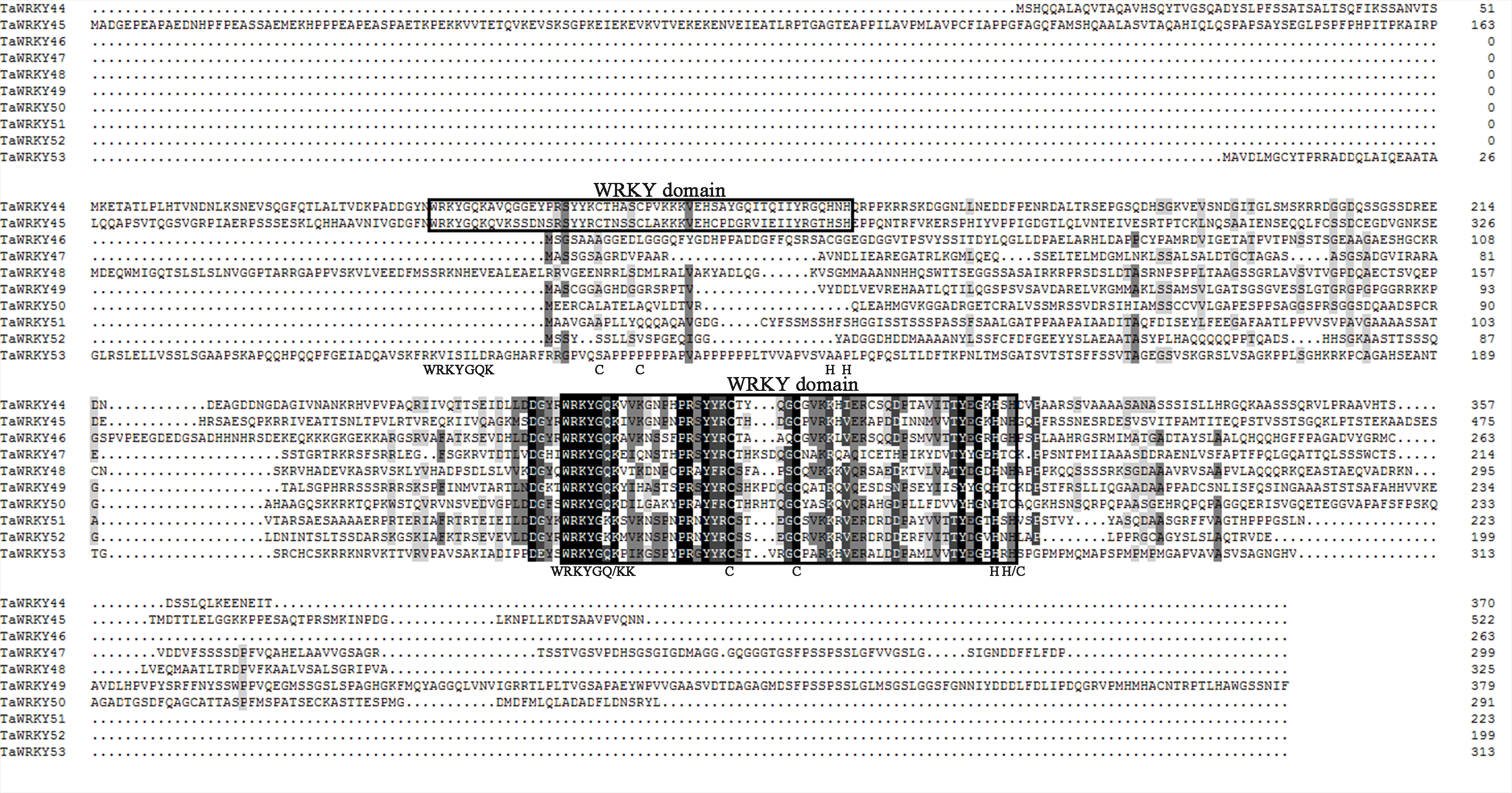

Supplement: Supplementary Figure 1 — Multiple alignments of the deduced amino acid sequences of TaWRKY44-TaWRKY53. [file Image1.TIF]

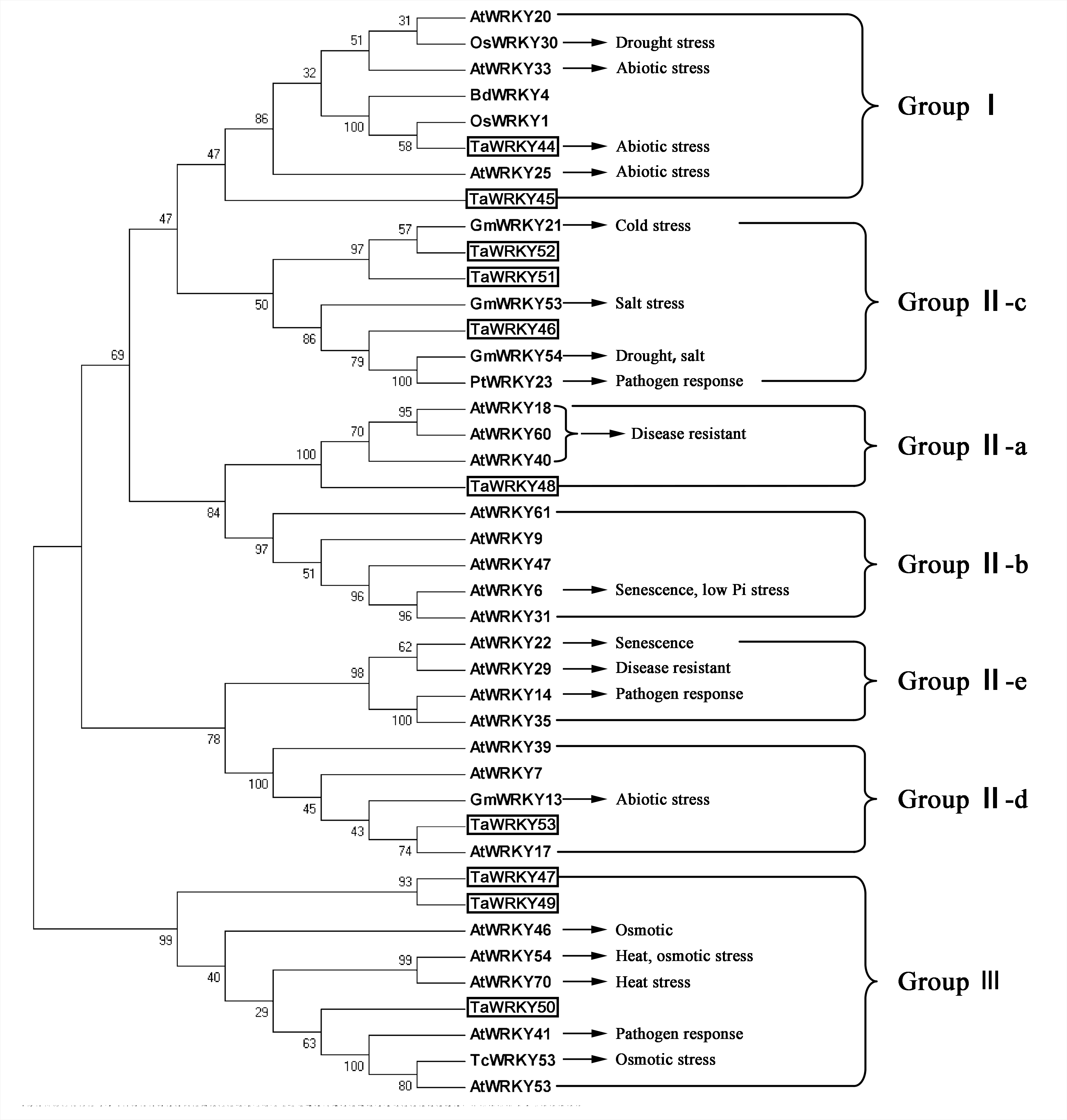

Supplement: Supplementary Figure 2 — Phylogenetic analysis of TaWRKY domains from various plants. [file Image2.TIF]
